# Draft Genome of the *Arthrobacter* sp. Strain Edens01

**DOI:** 10.1128/genomeA.01475-15

**Published:** 2015-12-17

**Authors:** M. B. Couger, Radwa A. Hanafy, Curtis Edens, Connie Budd, Donald P. French, Wouter D. Hoff, Mostafa S. Elshahed, Noha H. Youssef

**Affiliations:** a Department of Microbiology and Molecular Genetics, Oklahoma State University, Stillwater, Oklahoma, USA; b Department of Integrative Biology, Oklahoma State University, Stillwater, Oklahoma, USA

## Abstract

We report the draft genome sequence of *Arthrobacter* sp. strain Edens01, isolated from a leaf surface of a Rosa hybrid plant as part of the Howard Hughes Medical Institute-funded Student Initiated Microbial Discovery (SIMD) project. The genome has a total size of 3,639,179 bp and contig *N*_50_ of 454,897 bp.

## GENOME ANNOUNCEMENT

*Arthrobacter*, a bacterial genus frequently encountered in soil, has the capacity to metabolize numerous recalcitrant compounds (1), and is hence a valuable contributor to natural-attenuation-based, and engineered bioremediation schemes in multiple environments including groundwater (2, 3), crude-oil fields (4), and soil (5). Compounds that have been demonstrated to be metabolized by *Arthrobacter* spp. include chlorophenols (6), commonly found in pesticides, atrazine (7), a commonly used herbicide, nitrobenzoates (8), used in chemical and dye synthesis, and phthalates (9), compounds commonly used as a softening agent in plastics. Here, we present the genome of *Arthrobacter* sp. strain Edens01, which contains numerous genes important for bioremediation.

*Arthrobacter* sp. strain Edens01 was isolated from the leaf surface of a Rosa hybrid plant and sequenced at the University of Georgia Genomics Facility using the Illumina MiSeq sequencing platform, and 300 × 2 paired-end chemistry. Reads were quality-filtered with standard Illumina filtering settings, resulting in 753,618 read pairs, 452.2 MB of quality sequence data. All quality-filtered reads were assembled using the short read de Brujin graph assembly (10) program Velvet (11) with set to a k-mer value of 101 bp and a minimum contig coverage value of 7×. Gene models were created using the prokaryotic gene calling software package Prodigal (12). The Velvet assembly had a total size of 3,639,179 bp, a G+C content of 64.73%, and 3,374 predicted proteins. Translated protein sequences were functionally annotated using a combination of NCBI Blast C++ homology search (13) and HMMER 3.0 hmmscan (14) against the PFAM 26.0 database (15).

Based on 16S rRNA gene-based comparisons to genomes publicly available in GenBank database (*n* = 302,955,543, October 2015), strain Edens01 was most closely related (97.0% sequence similarity) to *Arthrobacter* sp. 35W genomic scaffold K254DRAFT (GenBank accession number NZ_AXVQ01000000). *Arthrobacter* sp. strain Edens01 16S rRNA gene also shared 97.0% sequence similarity with *Arthrobacter* sp. Rue61a, *Arthrobacter aurescens* TC1, *Arthrobacter* sp. M2012083, *Arthrobacter* sp. H41, *Arthrobacter* sp. 31Y, *Arthrobacter* sp. 135MFCol5, *Arthrobacter* sp. Br18, *Arthrobacter* sp. CAL618, and *Arthrobacter* sp. TB 23. Whole-genome comparison of *Arthrobacter* sp. Edens01 to protein coding gene models in the related genomes using BLASTp (e^−5^ cutoff) revealed a high proportion of shared genes (core genome) between *Arthrobacter* sp. Edens01 and closely related *Arthrobacter* spp. (2,703/3,374, 80.1% with strain Rue61a, 2,702/3,374, 80.1% with *Arthrobacter aurescens* TC1, 2,715/3,374, 81.9% with *Arthrobacter* sp. M2012083, 2,402/3,374, 71.2% with *Arthrobacter* sp. H41, 2,645/3,374, 78.4% with *Arthrobacter* sp. 135MFCol5, 2,482/3,374, 73.6% with *Arthrobacter* sp. Br18, 2,498/3,374, 74.0% with *Arthrobacter* sp. CAL618, and 2,536/3,374, 75.2% with *Arthrobacter* sp. TB 23). Genomic analysis of *Arthrobacter* sp. Edens01 revealed numerous genes putatively involved in the degradation of monoaromatics and xenobiotics, including protocatechuate 3,4-dioxygenase (16), phenol 2-monooxygenase (17), 4-hydroxybenzoate 3-monooxygenase (18), ethyl tert-butyl ether degradation protein (19), and pentachlorophenol 4-monooxygenase (20).

In conclusion, this initial genomic analysis of strain Edens01 reveals the presence of many genes involved in bioremediation and contributes to the study and pangenomic repertoire of the metabolically versatile genus *Arthrobacter*.

### Nucleotide sequence accession number.

The GenBank accession number for the genome is LKIU00000000.
